# Supporting maintenance of sugar-sweetened beverage reduction using automated versus live telephone support: findings from a randomized control trial

**DOI:** 10.1186/s12966-018-0728-7

**Published:** 2018-10-04

**Authors:** Jamie M. Zoellner, Wen You, Paul A. Estabrooks, Yvonnes Chen, Brenda M. Davy, Kathleen J. Porter, Valisa E. Hedrick, Angela Bailey, Natalie Kružliaková

**Affiliations:** 1 Department of Agricultural and Applied Economics, Virginia Tech, Blacksburg, VA 24061 USA; 2 Department of Health Promotion, University of Nebraska Medical Center, Omaha, NE 68198 USA; 3 School of Journalism, University of Kansas, Lawrence, KS 66045 USA; 4 Department of Human Nutrition, Foods and Exercise, Virginia Tech, Blacksburg, VA 24061 USA; 5 Department of Movement Arts, Health Promotion & Leisure Studies, Bridgewater State University, Bridgewater, MA 02325 USA; 6 Department of Public Health Sciences, School of Medicine, University of Virginia, P.O. Box 800717, Charlottesville, VA 22908-0717 USA; 7 Cancer Center without Walls at the UVA Cancer Center, 16 East Main St, Christiansburg, VA 24073 USA

**Keywords:** Beverages, Randomized controlled trial, Maintenance, Rural population, Behavioral research, Technology

## Abstract

**Background:**

Although reducing sugar-sweetened beverage (SSB) intake is an important behavioral strategy to improve health, no known SSB-focused behavioral trial has examined maintenance of SSB behaviors after an initial reduction. Guided by the RE-AIM framework, this study examines 6–18 month and 0–18 month individual-level maintenance outcomes from an SSB reduction trial conducted in a medically-underserved, rural Appalachia region of Virginia. Reach and implementation indicators are also reported.

**Methods:**

Following completion of a 6-month, multi-component, behavioral RCT to reduce SSB intake (SIP*smart*ER condition vs. comparison condition), participants were further randomized to one of three 12-month maintenance conditions. Each condition included monthly telephone calls, but varied in mode and content: 1) interactive voice response (IVR) behavior support, 2) human-delivered behavior support, or 3) IVR control condition. Assessments included the Beverage Intake Questionnaire (BEVQ-15), weight, BMI, and quality of life. Call completion rates and costs were tracked. Analysis included descriptive statistics and multilevel mixed-effects linear regression models using intent-to-treat procedures.

**Results:**

Of 301 subjects enrolled in the 6-month RCT, 242 (80%) were randomized into the maintenance phase and 235 (78%) included in the analyses. SIP*smart*ER participants maintained significant 0–18 month decreases in SSB. For SSB, weight, BMI and quality of life, there were no significant 6–18 month changes among SIP*smart*ER participants, indicating post-program maintenance. The IVR-behavior participants reported greater reductions in SSB kcals/day during the 6–18 month maintenance phase, compared to the IVR control participants (− 98 SSB kcals/day, 95% CI = − 196, − 0.55, *p* < 0.05); yet the human-delivered behavior condition was not significantly different from either the IVR-behavior condition (27 SSB kcals/day, 95% CI = − 69, 125) or IVR control condition (− 70 SSB kcals/day, 95% CI = − 209, 64). Call completion rates were similar across maintenance conditions (4.2–4.6 out of 11 calls); however, loss to follow-up was greatest in the IVR control condition. Approximated costs of IVR and human-delivered calls were remarkably similar (i.e., $3.15/participant/month or $38/participant total for the 12-month maintenance phase), yet implications for scalability and sustainability differ.

**Conclusion:**

Overall, SIP*smart*ER participants maintained improvements in SSB behaviors. Using IVR to support SSB behaviors is effective and may offer advantages as a scalable maintenance strategy for real-world systems in rural regions to address excessive SSB consumption.

**Trial registry:**

Clinicaltrials.gov; NCT02193009; Registered 11 July 2014. Retrospectively registered.

## Background

Health concerns surrounding the excessive consumption of sugar-sweetened beverage (SSB) intake is arguably one of the most publicized and controversial topics among current public health issues. SSB currently contributes approximately 7% of total energy intake for United States adults [1]; and rank third in overall food sources of energy and rank first in sources of carbohydrate [2]. Of additional importance are the significantly higher SSB intakes among rural adults [3] and the inverse relationship between intake of added sugars and educational attainment [4]. For example, in the Appalachian region targeted by this research, residents consume over three times the national average of SSB [5]. Our Appalachian data suggest overall consumption of added sugar comprises an estimated 21% of total energy intake among adults [6]; and, similar to national data, SSB is the largest contributor to added sugar intake in this region.

There are strong scientific data indicating associations among SSB and numerous health issues such as obesity, type 2 diabetes, cardiovascular disease, caries and oral health [7–11]. There is also extensive debate regarding the proposed solutions to excessive SSB intake. When compared to macro-level approaches to reduce SSB consumption [12–15], behavioral programs are generally viewed as much more acceptable, met with less political opposition, and regarded as a necessary complement to any higher level (e.g., community, policy) strategy. However, effective behavioral interventions targeting SSB reduction are only useful if post-program behavior changes are maintained and if real-world systems can implement and sustain the programs. Although attaining and maintaining current recommendations for SSB intake over long periods of time is an important behavioral strategy to improve health, no known SSB-focused behavioral trial has examined maintenance of SSB behaviors [16].

Maintenance, at the individual level, refers to the long-term effects of a program on outcomes six or more months after the most recent intervention contact [17]. Behavioral maintenance represents a key challenge for the prevention and treatment of chronic disease [18]. Likewise, maintenance of change following interventions is not often reported, especially in community-based interventions [19, 20]. Extended care provides prolonged participant contact to prevent relapse following initial behavior change and is a viable strategy to address long-term maintenance of health behaviors following an intervention [21]. Telephone-delivered extended care programs represent a potentially effective and low-cost way for promoting long-term health behavior change in rural communities [21, 22].

Though little is known about maintenance effects and use of extended care strategies to support SSB behaviors, the weight loss literature provides some useful insight. The use of extended care is recommended to address the issue of maintenance [21]; however, a systematic review of the effectiveness of technology-based (internet, telephone and interactive television) weight-loss maintenance interventions found mixed results [23]. Overall, the review found technology based extended care was more effective than usual care, but not as effective as personal contact. Nonetheless, when considering extended care programs in rural communities, it is necessary to explore different delivery methods because of increased travel to reach participants and potential costs associated with personal contact for both the delivery system and participants [24, 25].

Automated telephone calls utilizing interactive voice response (IVR) systems may represent a cost-effective and acceptable strategy to facilitate ongoing engagement in SSB and other health behaviors with individuals in rural areas [26, 27]. Several studies support the use of IVR calls for chronic disease self-management [28], physical activity promotion [29, 30], and smoking cessation [30]. However, there is limited research related to the use of IVR delivered telephone calls as a maintenance strategy, especially when compared to human-delivered telephone calls [23, 31]. Likewise, there is a dearth of behavioral maintenance interventions that compare human to automated strategies and also examine cost and scalability, or the capacity of a system or process to accommodate a growth in the number of participants.

In addition to examining individual level maintenance, it is also critical to understand reach and implementation dimensions as these factors influence the potential of real-world systems sustaining extended care strategies. The RE-AIM (reach, effectiveness, adoption, implementation, maintenance) framework helps guide the evaluation of behavioral interventions, highlighting the importance of both internal and external validity elements [32]. Evidenced across numerous systematic RE-AIM reviews, information on implementation indicators are typically underreported, including information on intervention cost [33–35]. Combined with the challenges of individual-level behavioral maintenance, there is also a gap in the literature around the cost and scalability of effective maintenance strategies [36, 37]. This gap is even wider in rural underserved regions, where behavioral and health disparities persist.

SIP*smart*ER is a theory-based, 6-month, multi-component health literacy intervention designed to reduce SSB intake among rural Appalachian adults [38]. Using a randomized controlled trial (RCT), SIP*smart*ER has been shown to be effective from baseline to 6-months. Relative to matched-contact comparison group targeting physical activity behaviors (e.g. MoveMore), SIP*smart*ER participants significantly decreased SSB intake, improved overall dietary and beverage quality, and demonstrated improvement in a δ^13^C added sugar intake biomarker [39–41]. SIP*smart*ER also yielded small, yet significant, improvements in weight and BMI. At completion of the 6-month intervention, participants were further randomized to one of three 12-month maintenance conditions (i.e., behavior-specific IVR calls, behavior-specific human-delivered support calls, IVR call control condition) [38]. Data from this maintenance phase have not yet been reported.

Guided by the RE-AIM evaluation framework, this manuscript focuses on the reach, maintenance of effects, and implementation of maintenance. The primary aim of this manuscript is to explore individual-level maintenance of outcomes. As such, the first objective is to compare 0–18 and 6–18 month outcomes [i.e., SSB intake, weight, BMI, quality of life (QOL)] between SIP*smart*ER participants and matched-contact comparison participants, regardless of randomized maintenance condition. We hypothesized that 0–6 month improvements in SSB intake, weight, BMI, and QOL would be sustained in the 12-month maintenance phase for SIP*smart*ER participants. The second objective is to examine individual-level outcomes by randomized maintenance condition among SIP*smart*ER participants. We hypothesize that maintenance effects for both the behavior-specific IVR calls and behavior-specific human-delivered support calls would be superior when compared to the IVR call control condition. Secondary aims are to explore reach and implementation indicators of the maintenance phase, including call completion rates and costs.

## Methods

This RCT took place between 2012 and 2015 and occurred in eight southwestern Virginian counties. These targeted rural, Appalachia counties are federally designated as a medically underserved area [42] and have an average rurality status of 6.3 on the 9-point Rural-Urban Continuum Codes (1 = urban, 9 = completely rural) [43]. Similarly, these counties consistently score lowest on the Health Opportunity Index (i.e., less opportunity) [44].

### Ethics approval

The Virginia Tech Institutional Review board approved all study procedures. Participants were informed of the random allocation process and provided written consent to participate. Gift cards were provided at the baseline, 6-month and 18-month assessments (i.e., $25, $50, and $75, respectively).

### Study design, eligibility & intervention descriptions

**Fig. 1:**
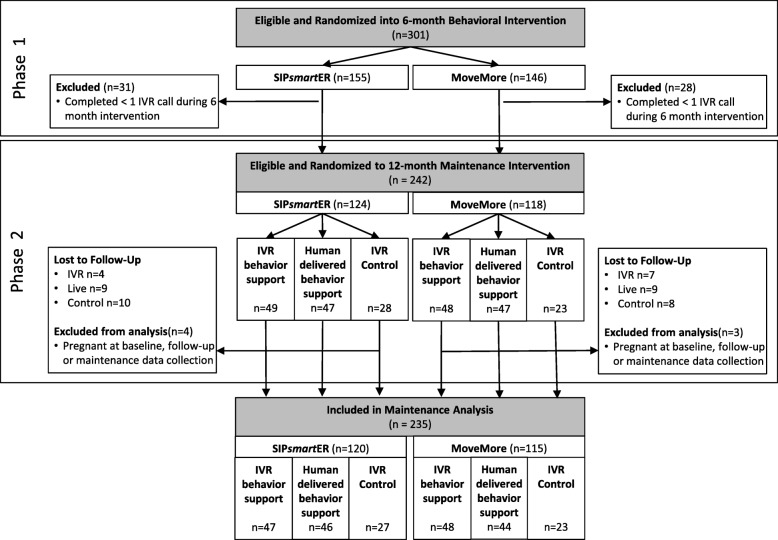
CONSORT diagram of participant randomization and flow

The consort diagram illustrates participant flow and randomization **(**Fig. 1). As described below, this 2-phased study included two time points of randomization.

#### Phase 1, 6-month behavioral intervention

In Phase 1, and using a simple randomization protocol, eligible participants were randomly assigned to the SIP*smart*ER (*n* = 155) or MoveMore comparison condition (*n* = 146). Eligibility criteria for Phase 1 enrollment included English-speaking adults who were 18 years of age or older, consumed > 200 SSB kcals/day, reported no contraindications for physical activity, had regular access to a telephone, and who were not concurrently enrolled in another nutrition or physical activity program. Throughout the targeted counties a variety of active (e.g., recruitment at health departments) and passive (e.g., flyers, newspaper ads, word of mouth) recruitment strategies were used. Full recruitment, reach, and representativeness data are presented elsewhere [45].

Both the SIP*smart*ER and MoveMore conditions were 6-month behavioral modification programs that included three small group sessions, one teach-back call, 11 IVR telephone calls, completion of personalized action plans and self-monitoring log sheets. A detailed account of the structure, theoretical constructs, and content of the classes and IVR calls is described elsewhere [38, 46, 47]. Both conditions were guided by Theory of Planned Behavior and health literacy concepts and strategies, and were designed for broad dissemination [48–52].

- *SIPsmartER condition.* Individuals enrolled in SIP*smart*ER participated in a 6-month behavioral modification program aimed to decrease SSB intake, with the primary goal of achieving the SSB recommendation of less than 8 fluid ounces per day [53, 54]. SIP*smart*ER was the main program of interest in this RCT and SSB behavior was the primary outcome. In this maintenance manuscript, we also mainly focus on the outcome of the SIP*smart*ER condition, and use MoveMore as the comparison condition in applicable hypothesis driven analysis.
- *MoveMore condition.* Individuals enrolled in MoveMore participated in a 6-month behavioral modification program aimed to increase physical activity, with the primary goal of achieving 150 min of moderate intensity PA and muscle strengthening activities on two or more days per week.

During Phase 1, when two subsequent intervention activities were missed, a research staff member would attempt to re-engage the participant with a live telephone call to complete the content of the missed activity (i.e. class and/or IVR call) using a semi-structured script. Average IVR call completion rate was 51% during Phase 1 [40]. Even though the IVR support component was designed for automation and scalability, a substantial number of calls during Phase 1 were attempted and delivered by a research team member. This finding, along with maintenance literature indicating that technology based extended care were not as effective as personal contact [23], informed the Phase 2 maintenance conditions.

#### Phase 2, 12-month maintenance period

In Phase 2, and the focus of this manuscript, participants were further randomized to one of three 12-month maintenance conditions. Each condition included monthly telephone calls, but varied in mode and content: 1) IVR behavior support, 2) human-delivered behavior support, or 3) IVR control condition. Eligibility criteria for Phase 2 included enrollment in Phase 1 and completion of at least one IVR call during the initial 6-month intervention. Furthermore, Phase 2 participants were stratified into four groups based on the following criteria: a) completed 6-month assessment and completed < 6 IVR calls (i.e. < 50% calls), b) completed 6 month assessment and completed > 6 IVR calls, c) did not complete 6 month assessment and completed < 6 IVR calls or d) did not complete 6 month assessment and completed > 6 IVR calls). Subsequently, randomization into a maintenance condition occurred within each stratum. This process promoted a balanced prognosis for call participation within each of the maintenance conditions. Within the SIP*smart*ER and MoveMore comparison condition, the planned allocation ratio was 2:2:1, respectively, for IVR behavior support, human-delivered behavior support, and IVR control condition.

- *IVR behavioral support condition.* Participants in this group received TPB informed telephone support calls from an automated IVR system. The 11 monthly calls were structurally similar to the 11 IVR calls received in the initial 6-month intervention [38, 46]. During each maintenance call, participants reported their behavior (SIP*smart*ER reported ounces of SSB; MoveMore reported minutes of PA), received tailored feedback based on goal maintenance and could elect to set a new maintenance goal. Participants then either identified new barriers and strategies pertaining to their behavior or kept the same ones identified previously. The call ended with a short TPB-based message that reinforced key information presented during Phase 1.
- *Human-delivered behavioral support condition.* Participants in this group received TPB-based telephone support calls that included 11 monthly calls from an appointed member of the research team. The scripted call followed an identical format as the IVR behavioral condition described above.
- *IVR control condition.* Participants in the control group received 11 monthly IVR calls that included monthly updates on the study such as, “it’s been three months since we’ve seen you,” and delivered entertaining science facts such as, “sunshine can help your sleep patterns.” Participants did not report their current behavior, set goals, or hear a TPB-based support message. Additionally, information specific to SSB or PA was not addressed in the control call.

In both the IVR and human-delivered behavioral support conditions, maintenance calls were attempted three times, either by the IVR system or by a research assistant. After the third attempt, the call was categorized as not completed and no further attempts were made to reach the participant that month. To promote retention at the 18-month assessment time, all participants received a birthday card and a season’s greeting card during the maintenance phase.

### Measures

Eligibility and demographic information was collected during the screening process prior to enrollment in Phase 1. The screening instrument included questions about sex, age, race/ethnicity, education level, family/household income, employment status, health care coverage, marital status, number of children in the home, county of residence, SSB intake and contraindications for physical activity. Health literacy was assessed using the Newest Vital Sign at baseline [55].

All outcomes were measured during health screenings in community-based settings (e.g., public health buildings, Extension offices, churches) that occurred at baseline, 6-months and 18-months. The primary outcome, SSB intake, was measured by the BEVQ-15, a validated food-frequency instrument that assesses beverage consumption over the past month [56]. SSB intake is calculated by summing five items including regular soft drinks, sweetened juice beverage/drink, sweetened tea, coffee with sugar, and energy and sports drinks. Weight was measured without shoes and light clothing using a calibrated digital Tanita scale (Model: 310GS). Height was measured with a research-grade stadiometer. The Centers for Disease Control Healthy Days module was used to assess quality of life [57].

Implementation was defined as the number of telephone calls completed over the 12-month maintenance intervention (out of a possible 11). Bi-monthly research team meetings and on-line tracking forms were used to monitor fidelity to established protocols, including human-delivered behavior calls attempts and completion. For the IVR calls, records were maintained related to the cost of hosting the platform during the duration of the maintenance phase. Implementation costs of the maintenance conditions were tracked through monthly financial records, including invoices from our IRV vendor, as well as research assistants’ salary information and call attempt and time log records.

### Statistical analysis

All data were entered into SPSS statistical analysis software (version 22.0, 2012, International Business Machines Corporation, Pittsburgh, PA) and validated scoring procedures were applied to compute outcome variable scores. Descriptive statistics were used to summarize demographic characteristics and participation rates [e.g. means, standard deviations (SD), medians and interquartile range (IQR)], as well as costs. Chi square tests of association or Fisher’s exact tests (categorical variables) and ANOVA (continuous variables) were used to compare demographics between participants enrolled and not enrolled in Phase 2 and to examine differences in engagement rates among randomized conditions.

Multilevel mixed-effects linear regressions analyses were performed using Stata software to account for clustering of individuals within county cohorts (version 13, 2013, StataCorp LP, College Station, TX). The mixed-effect models controlled for individual baseline characteristics, dummies of time and condition, and a time by condition dummy interaction. All models calculated robust standard errors for county/cohort cluster. The baseline covariates controlled in the models were chosen a priori and included age, gender, race/ethnicity, income, education level, health literacy level, employment status, number of children, smoking status, and BMI [58]. For analytical purposes, all women who were pregnant at any study point were excluded from the maintenance analysis.

Our initial analyses included 18-month present at follow-up (completers only), as well as intention-to-treat using last-observation-carried-forward. These analytic procedures yielded similar results, with the expected largest effect sizes found in the present at follow-up analysis. Therefore, intention-to-treat results, the more conservative effects, are reported in this manuscript [59, 60]. We also report Cohen’s d effect sizes for the relative mean treatment effects between the randomized conditions [61].

This trial was powered to detect a small effect size of 0.34 for 0–6 month changes in SSB intake between the SIP*smart*ER and MoveMore conditions (i.e., 80% power, 0.05 type 1 error). Since this trial was not specifically designed or powered to detect maintenance effects, the analyses and interpretation of findings are considered exploratory.

## Results

### Reach

Of 301 subjects enrolled in Phase 1, 242 (80.4%) were further randomized in the maintenance phase (Fig. 1). Of the 124 SIP*smart*ER participants in the maintenance phase, randomization included 49 in the IVR-behavior support condition, 47 in the human-delivered behavior support condition, and 28 in the IVR control condition. Of the 118 MoveMore participants in the maintenance phase, randomization included, 48 in the IVR-behavior support condition, 47 in the human-delivered behavior support condition, and 23 in the IVR control condition.

Of those randomized in the maintenance phase, 57 were lost to follow-up and 195 (81%) returned for the 18-month assessment. The loss to follow up between SIP*smart*ER (23 of 124; 19%) and MoveMore (24 of 118; 20%) conditions were similar. However, the loss to follow up was greatest for the IVR control group (18 of 51; 35%), followed by the human-delivered behavior support condition (18 of 94; 19%), and was lowest among the IVR behavioral support condition (11 of 97; 11%). Seven pregnant women (i.e., four in SIP*smart*ER and three in MoveMore) were excluded from all additional maintenance phase analyses, resulting in 235 participants.

Table 1 illustrates the demographic characteristics of participants included in this maintenance phase analysis, including the 120 SIP*smart*ER and 115 MoveMore participants. The majority of the participants were aged 25–64, female, Caucasian, and had an annual household income <$35,000. The average baseline BMI was around 33, with 22% classified as overweight and 57% as obese. To describe representativeness, Table 1 also explores differences among participants enrolled in the maintenance phase to those originally enrolled in the trial but not into the maintenance phase. In the SIP*smart*ER condition, compared to those who were not randomized into the maintenance phase, those included in the maintenance phase were older and more likely to be classified as unable to work/on disability. In the MoveMore comparison condition, there were no significant demographic difference between those who enrolled and did not enroll in the maintenance phase.

**Table 1:** Baseline characteristics of participants who had participated in 6-month behavioral modification program, by randomized condition assignment, and representativeness of enrolled maintenance sample (*n* = 235)

Characteristics	SIP*smart*ER			MoveMore		
	Maintenance sample (*n* = 120)^a^	Not Enrolled in maintenance (*n* = 31)^a^	Test statistic *p*-value	Maintenance sample (*n* = 115)^a^	Not Enrolled in maintenance (*n* = 28)^a^	Test statistic *p*-value
Age						
Age (years), M (SD)	44.2 (12.6)	32.1 (12.1)	F = 23.0 *p* = ≤0.001	41.6 (13.1)	46.5 (13.7)	F = 3.0 *p* = 0.08
Age 18–24	11 (9)	11 (35.5)	χ^2^ = 15.3 *p* = 0.002	12 (10)	2 (7)	χ^2^ = 3.9 *p* = 0.27
Age 25–44	54 (45)	13 (42)		57 (50)	9 (32)	
Age 45–64	52 (43)	7 (22.5)		43 (37)	16 (57)	
Age ≥ 65	3 (3)	0 (0)		3 (3)	1 (4)	
Sex						
Male	24 (20)	6 (19)	χ^2^ = 0.006 *p* = 0.94	20 (17)	6 (21)	χ^2^ = 0.25 *p* = 0.62
Female	96 (80)	25 (81)		95 (83)	22 (79)	
Race						
Caucasian	110 (92)	27 (87)	χ^2^ = 0.6 *p* = 0.43^b^	110 (96)	26 (93)	χ^2^ = 0.38 *p* = 0.54^b^
African American	9 (8)	1 (3)		3 (3)	0 (0)	
More than one race	1 (1)	2 (7)		2 (2)	2 (7)	
Other	0 (0)	1 (3)		0 (0)	0 (0)	
Ethnicity						
Hispanic/Latina	0 (0)	2 (7)	NA	1 (1)	0 (0)	NA
Annual Family/Household Income						
≤ 14,999	51 (43)	18 (58)	χ^2^ = 3.4 *p* = 0.33	43 (37.5)	13 (47)	χ^2^ = 1.6 *p* = 0.66
15,000-34,999	42 (35)	10 (32)		36 (31)	6 (22)	
35,000-54,999	16 (13)	2 (7)		16 (14)	5 (18)	
≥ 55,000	11 (9)	1 (3)		20 (17.5)	4 (14)	
Employment status						
Employed full or part time	55 (46)	15 (48)	χ^2^ = 5.1 *p* = 0.024	66 (57)	15 (54)	χ^2^ = 0.8 *p* = 0.38
Unable to work/on disability	29 (24)	1 (3)		13 (11)	5 (18)	
Number of children						
At least 1 child in household	58 (48)	19 (61)	χ^2^ = 1.7 *p* = 0.20	57 (50)	14 (50)	χ^2^ = 0.002 *p* = 0.97
No children in household	62 (52)	12 (39)		58 (50)	14 (50)	
Education level						
</=High school graduate	39 (32.5)	10 (32)	χ^2^ = 0.001 *p* = 0.98	33 (29)	11 (39)	χ^2^ = 0.12 *p* = 0.28
Some college or greater	81 (67.5)	21 (68)		82 (71)	17 (61)	
Anthropometry						
Weight (kg), M (SD)	91.8 (25.5)	86.2 (29.8)	F = 1.12 *p* = 0.29	92.5 (24.1)	83.7 (23.5)	F = 3.1 p = 0.08
BMI (kg/m^2^), M (SD)	33.7 (8.8)	31.5 (10.8)	F = 1.3 *p* = 0.25	33.3 (8.8)	31.0 (9.4)	F = 1.4 *p* = 0.24
Current Smoker	38 (32)	15 (48)	χ^2^ = 3.0 *p* = 0.08	30 (26)	10 (36)	χ^2^ = 1.0 *p* = 0.31
Health literacy (HL) status^c^						
High likelihood or Possibility of limited HL (score 0–3)	46 (38)	9 (29)	χ^2^ = 0.9 *p* = 0.34	31 (27)	10 (36)	χ^2^ = 0.8 *p* = 0.36
Adequate HL (score 4–6)	74 (62)	22 (71)		84 (73)	18 (64)	
Baseline SSB, kcals	495 (401)	500 (254)	F = 0.004 *p* = 0.95	390 (289)	347 (279)	F = 0.35 *p* = 0.56

F test were used to compare means across conditions and chi square tests were used to compare proportions across the conditions. Cells do not always equal 100% due to rounding

*M* = Mean, *SD* = Standard Deviation, *NA* = not applicable due to small cell count

^a^*n* (%) unless otherwise noted

^b^Due to small cell count, reported χ^2^ statistic represents Caucasian compared to all other races

^c^Health literacy was assessed using the validated Newest Vital Sign

### Maintenance of effects

**Fig. 2:**
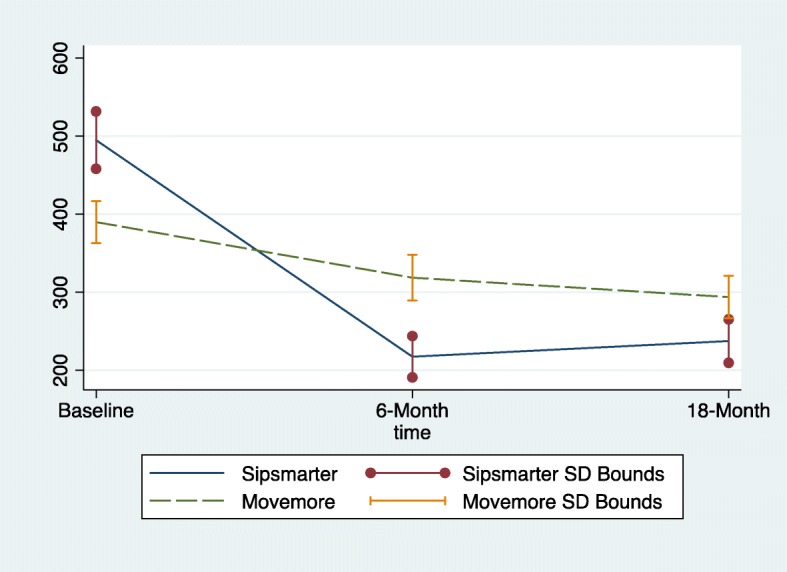
Average sugar-sweetened beverage (SSB) kcals at baseline, 6-months and 18-months, by SIPsmartER versus MoveMore conditions

Table 2 describes the adjusted changes in 0–6 months, 6–18 months, and 0–18 months outcomes and relative effects between the SIP*smart*ER and MoveMore comparison conditions, regardless of randomized maintenance condition. SIP*smart*ER participants maintained significant 0–18 month decreases in SSB intake by 256 (95% CI = − 339, − 174, *p* < 0.01) kcals/day when compared to the 96 (95% CI = − 149, − 43, *p* < 0.01) kcals/day decrease among the MoveMore comparison participants (*p* < 0.01)(Cohen’s d effect size = 0.47) (Fig. 2). The 0–18 month relative effects between conditions for weight and BMI changes were not significant, yet the magnitude of change for SIP*smart*ER was similar to the 0–6 month outcomes. The trends for improvements in outcomes among the MoveMore comparison condition in the 6–18 month maintenance phase, though not significant, is noteworthy and has implications for interpreting between condition effects. For SSB, weight, BMI and quality of life outcomes, the 6–18 month changes were not significant, implying a maintenance of the effects achieved at conclusion of Phase 1 among SIP*smart*ER participants.

**Table 2:** Adjusted 6- and 18-months outcomes and relative effects between SIP*smart*ER (*n* = 120) and MoveMore (*n* = 115) conditions

Outcome	Condition	Baseline^a^	Δ, baseline to 6 months^b^	Relative 0 to 6 month effects between conditions^b,c^	Δ, 6 to 18 months^b^	Relative 6 to 18 month effects between conditions^b,c^	Δ, baseline to 18 months^b^	Relative 0 to 18 month effects between conditions^b,c^
SSB, kcals	SS	495 (401)	− 277 (− 375, − 179)***	−206 (− 309, − 102)***	21 (−13,54)	46 (− 13, 105)	−256 (− 339, − 174)***	−160 (− 264, − 56)**
	MM	390 (289)	−71 (− 112, − 31)***		−25 (− 66,16)		− 96 (− 149, − 43)***	
BMI, kg/m^2^	SS	33.71 (8.84)	−0.26 (− 0.44, − 0.09)**	−0.32 (− 0.66, 0.02)	0.05 (− 0.31, 0.42)	0.24 (− 0.11, 0.58)	−0.21 (− 0.71, 0.30)	−0.08 (− 0.60, 0.44)
	MM	33.28 (8.85)	0.06 (−0.21, 0.33)		−0.18 (− 0.72, 0.35)		− 0.12 (− 0.70, 0.45)	
Weight, kg	SS	91.8 (25.5)	−0.58 (− 1.13, − 0.04)*	−0.54 (− 1.29, 0.21)	0.03 (− 0.74, 0.80)	0.44 (− 0.39, 1.28)	−0.55 (− 1.64, 0.53)	−0.10 (− 1.22, 1.03)
	MM	92.54 (24.1)	−0.04 (− 0.48, 0.40)		− 0.42 (− 1.79, 0.96)		−0.46 (− 1.93, 1.02)	
QOL, # of unhealthy days	SS	8.8 (9.5)	−1.7 (− 2.9, −0.4)**	−1.5 (− 2.8, − 0.3)*	0.5 (− 0.6, 1.6)	0.8 (− 0.9, 2.4)	−1.1 (− 3.3, 1.0)	−0.7 (− 2.9, 1.5)
	MM	7.4 (7.6)	−0.1 (− 1.0, 0.7)		− 0.3 (− 1.2, 0.6)		− 0.4 (− 1.6, 0.7)	

Within condition and between condition statistical significance indicated by asterisks: *p < 0.05, **p < 0.01, ***p < 0.001

*SS* = SIP*smart*ER condition, *MM* = MoveMore condition, *SSB*=Sugar-sweetened beverages, *BMI*=Body Mass Index, *QOL* = Quality of Life

^a^Means (Standard Deviations) are not adjusted for covariates

^b^Values reported as mean change (95% Confidence Interval). All Δ change scores represent values from the first (earliest) time point subtracted from later time point

^c^Condition dummy variable coded as SIP*smart*ER vs. MoveMore (base). Models controlled for baseline covariates including age, gender, race/ethnicity, income, education level, health literacy level, employment status, number of children, smoking status, and BMI. The 95% confidence intervals are also adjusted to be cohort robust. Analytic procedures use intention-to-treat last observation carried forward imputations and empirical models are multi-level mixed effect models

**Fig. 3:**
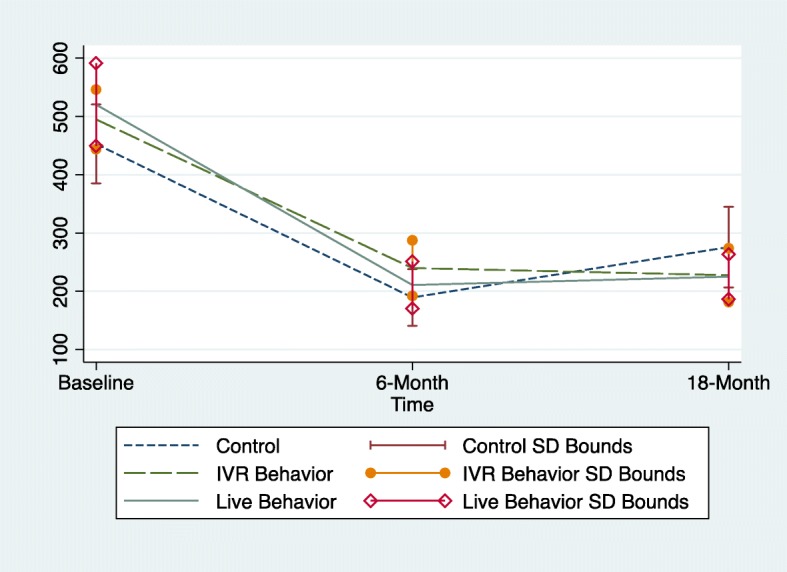
Average sugar-sweetened beverage (SSB) kcals at baseline, 6-months and 18-months among SIPsmartER participants, by randomized maintenance assignment

For the SIP*smart*ER condition only, adjusted changes in 6–18 month outcomes by randomized maintenance assignment are described in Table 3. Within each condition, there were no statistically significant 6–18 month changes for any of the outcomes. However, the IVR-behavior group reported significantly greater reductions in SSB kcals/day during the 6–18 month maintenance phase, compared to IVR control condition, (− 98, 95% CI = − 196, − 0.55) (*p* < 0.05) (Cohen’s d effect size = 0.42). There were no significant differences in SSB kcal/day between the human-delivered behavior support condition and IVR control condition (− 70, 95% CI = − 209, 64, NS) (Cohen’s d effect size = 0.25) or between the IVR-behavior vs human-delivered behavior conditions (27, 95% CI = − 69, 125, NS)(Cohen’s d effect size = 0.12) (Fig. 3). Finally, there were no significant differences among the randomized maintenance conditions for weight, BMI, or quality of life.

**Table 3:** Adjusted 6–18 months outcomes by randomized maintenance assignment in the SIP*smart*ER condition (*n* = 120)

	Change 6 to 18 months^a^			Relative 6 to 18 month effects between conditions^a,b^		
Outcome	IVR behavior *n* = 47	Human-delivered behavior *n* = 46	IVR control *n* = 27	IVR behavior vs. IVR control	Human-delivered behavior vs. IVR control	IVR behavior vs. Human-delivered behavior
SSB, kcals	−12.1 (− 65, 41)	15.6 (− 47, 78)	86 (− 19, 192)	− 98.5 (− 196, − 0.55)*	−70 (− 209, 64)	27.7 (− 69.6, 125.2)
BMI, kg/m^2^	0.11 (−0.28, 0.51)	0.07 (− 57, .70)	− 0.07 (− 0.60, 0.46)	0.18 (− 0.37, 0.74)	0.13 (− 0.49, 0.76)	−0.05 (− 0.80, 0.70)
Weight, kg	0.04 (− 0.97, 1.1)	−0.17 (− 1.4, 1.13)	0.36 (− 1.7, 2.44)	−0.32 (− 2.7, 2.1)	−0.53 (− 2.9, 1.88)	−0.21 (− 1.9, 1.47)
QOL, # of unhealthy days	0.6 (−1.7, 2.9)	− 0.7 (− 3.7, 2.3)	2.4 (− 0.6, 5.4)	−1.8 (− 5.1, 1.4)	−3.1 (− 8.3, 2.1)	−1.3 (− 5.7, 3.2)

Within condition and between condition statistical significance indicated by asterisks: *p < 0.05

*SSB*=Sugar-sweetened beverages, *BMI*=Body Mass Index, *QOL* = Quality of Life

^a^Values reported as mean change (95% Confidence Interval). All Δ change scores represent values from the first (earliest) time point subtracted from later time point

^b^Condition dummy variable coded as interactive voice response (IVR) behavior support vs. IVR control condition; human-delivered behavior support vs. IVR control condition; IVR behavior support vs. human-delivered behavior support. Models controlled for baseline covariates including age, gender, race/ethnicity, income, education level, health literacy level, employment status, number of children, smoking status, and BMI. The 95% confidence intervals are also adjusted to be cohort robust. Analytic procedures use intention-to-treat last observation carried forward imputations and empirical models are multi-level mixed effect models

### Implementation

Among all 235 participants in both the SIP*smart*ER and MoveMore conditions, call completion rates were not significantly different among randomized maintenance conditions. Of the 11 possible calls, average completion rates were 4.6 (SD = 4.5; Median = 4.0; IQR = 9.0) IVR-behavior calls, 4.4 (SD = 3.4; Median = 4.0; IQR = 5.0) human-delivered behavior calls, and 4.2 (SD = 4.2; Median = 2.5; IQR = 8.3) IVR control calls (F = 0.17, *p* = 0.85). Specific to the 120 SIP*smart*ER participants, average completion rates were also not significantly different (F = 1.52, *p* = 0.22), including 5.2 (SD = 4.4; Median = 5.0; IQR = 9.0) IVR-behavior calls, 4.0 (SD = 3.2; Median = 3.0; IQR = 5.5) human-delivered behavior calls, and 5.0 (SD = 4.1; Median = 4.0; IQR = 7.0) IVR control calls.

Of the 235 maintenance participants, 191 participants were enrolled in the IVR conditions. The average monthly cost of hosting the IVR system was $600/month or $7200 total per the 12 months. This equates to $3.14/participant per month or $37.68/participant total for the 12-month IVR maintenance condition.

A total of 94 participants were allocated in the human-delivered behavior condition. Verified through financial statements, research staff time was estimated at $25/h ($0.41/min). According to tracking records, it took on average 2.5 attempts to reach the participants. We used an estimate of 2 min each time a call was attempted, including preparation time of the research staff. The average length of the human-delivered behavior calls was estimated at 7 min, an amount of time similar to the length of the IVR calls (i.e., 6.9 + 1.9) minutes) [40]. Therefore, the costs incurred for the human-delivered behavior condition were estimated using the following approach: (1) attempts to reach participants on phone = 2.5 attempts * 2.5 min * $0.41 min * 94 total participants = $192.70, (2) once on the phone = 7 min * $0.41 min * 36 participants (average actually reached) = $103.32, (3) total per month = $296.02/month or $3552.24 total per the 12 months, (4) since 94 participants enrolled, this equates to $3.15/participant per month or $37.78/participant total for the 12-month human-delivered behavior maintenance condition.

As further addressed in the discussion, the cost of maintaining the IVR system is fixed. However, the cost of human-delivered behavior maintenance condition is variable.

## Discussion

Prior systematic reviews have defined maintenance of behavior change as a statistically significant between-groups difference in favor of the intervention group at the end of the intervention and at follow-up for at least one behavioral outcome [20]. Following this criteria, our trial demonstrated overall maintenance of effects for the primary SSB outcome among SIP*smart*ER participants. Given the link between SSB and numerous chronic health conditions (e.g., type 2 diabetes, cardiovascular disease, caries and oral health) [7–11], our finding establishes that an individual-level intervention integrating behavioral theory and health literacy concepts can maintain SSB reductions among residents in a high-risk rural region.

When considering the three maintenance conditions for the SIP*smart*ER participants, we found the IVR behavioral support condition offered statistically significant advantages over the IVR control group for the SSB outcome. This finding supports prior literature which suggests that extended care interventions delivered via telephonic approaches and with continued action planning and tailored feedback can promote maintenance in outcomes [62]. On the contrary, the human-support delivered maintenance calls were not significantly different than IVR control or IVR behavior. This finding can be compared to prior weight loss literature that suggests technology facilitated approaches are typically more effective than usual care, but not more effective than personal contact [23, 63, 64]. However, given our small sample size, findings should be interpreted somewhat cautiously. Nonetheless, the SSB outcome findings in this exploratory secondary analysis, in combination with the implementation findings, support the promise of behavioral maintenance strategies delivered via IVR in rural regions.

Given the design of this trial, interpreting the secondary BMI and weight outcomes is complex. We hypothesized that 0–6 month improvements in SSB intake, weight, and BMI outcomes would be sustained in the 12-month maintenance phase for SIP*smart*ER participants. Arguably, one could also hypothesize that the MoveMore comparison group participants could achieve and maintain weight loss. As previously described, this community-based research trial was designed to assess SSB as the primary outcome (not weight) and provide the potential for all enrolled participants to benefit. In the 0–6 month phase, the SIP*smart*ER group achieved small, yet significant, improvements in weight and BMI; however, the MoveMore comparison group did not experience similar improvements. In contrast, during the 6–18 month maintenance phase, the MoveMore comparison condition had trends for improving weight and BMI outcomes, though not significant, whereas the SIP*smart*ER group shows relatively little change. The study design is important to consider when interpreting the relative between condition maintenance effects. Regardless, from a clinical significance perspective, the overall average weight and BMI changes are modest [65]. From a public health perspective, additional efforts and a fully powered study is needed to understand how maintenance of SSB behaviors influences clinical outcomes, including weight. Though our exploratory maintenance study has limitations in the design and power for weight-related outcomes, our findings suggest that focusing solely on individual-level SSB behavior change is insufficient to achieve and maintain clinically significant improvements in weight.

Our findings on the relative costs of the maintenance conditions may not be surprising when considered through the lens of scalability. First, related to ongoing implementation costs, the cost of IVR system maintenance is fixed—once activated the monitoring cost is consistent over time and does not vary as additional participants are added unless additional monitoring and phone lines are necessary. Second, and in contrast, the cost of the human-delivered behavior maintenance condition are variable—each new participant increases costs at an incremental rate. When considered together, taking these interventions to scale in a community or clinical setting the automated system costs per participant go down with additional participants whereas the per participant cost of human-delivered systems remains the same. Two additional considerations related to cost are wage rates and start-up costs. We used actual rates of research assistant time. If the human-delivered calls were implemented in a practice setting, this rate could be variable if replaced with market wage rate of staff that would ultimately implement the intervention. Also, since the start-up costs for both interventions—training and quality assurance methods for staff implementing the human-delivered interventions and initial construction of the IVR system were incurred in the first 6-month phase of the trial, they were not included in our maintenance estimates—though they would surely be considered in the decision making processes for community or clinical organizations when making adoption decisions. Despite these considerations, implementation and maintenance costs are known to be one of the most underreported aspects of informing the translation of evidence-based programs into real-world practice settings [22, 36, 37]. Our goal was to address this gap in the literature, while being transparent about the multitude of factors to consider when estimating and interpreting costs to implement and maintain IVR versus human-delivered maintenance support calls.

Though call completion rates in our study were similar across conditions, the overall 40% uptake rate (4 to 5 out of 11 calls completed) remains lower than desired. However, we found that the retention rate for participants in the IVR-behavioral condition was superior to that of the human delivered behavioral support. This finding may appear to contradict other studies of technology-based maintenance interventions which report low utilization of technology [66, 67]. This finding may be explained by the fact that our IVR technology was introduced and applied at the start of the intervention, not solely introduced at the maintenance phase. However, utilization rates of in-person and other human-delivered interventions are also known to decrease over time [68]. Our study is unique in comparing retention rates between human and technology-delivered interventions and our findings may also be the result of focusing our intervention efforts on rural participants. Nonetheless, given the recent acceleration of e/m health options, additional focus on retention rates and developing and testing strategies that can be used to reduce attrition continues to be an area in need of future research.

The study has several limitations. First, the findings may have limited generalizability beyond the targeted region of rural southwest Virginia. Second, SSB outcomes are based on self-reported outcomes, yet a validated measure was used. Importantly, self-reported SSB outcomes from the main Phase 1 trial were also supported by improvement in a δ^13^C added sugar intake biomarker changes [39], which further supports the validity of self-reported changes in this manuscript. Third, the MoveMore comparison condition and lack of a true control condition influences our findings, especially interpretations of between condition effects for the secondary weight-related outcomes. Similarly, the lack of no contact control group should be considered when interpreting the maintenance findings. Fourth, since participants who did not complete any IVR calls were not further randomized into the maintenance phase, our adherence and effects may be somewhat inflated. Finally, our exploratory study was not specifically powered to determine maintenance effects, hence the findings should be interpreted somewhat cautiously. Nonetheless, our study provides key information needed to inform the sample size of future studies examining maintenance of SSB effects and differences between maintenance conditions. Our study limitations should be interpreted within context of the study strengths, including the targeted high need rural region, RCT design, and conservative analysis using intent-to-treat procedures.

This exploratory study reveals several key areas of future research. Given the persistent disparities in access to care in rural regions, there is a high need for additional research on technology-based maintenance interventions in underserved and geographically dispersed communities. Furthermore, the maintenance and extended care literature is rather complex, including multifaceted issues related to maintenance data interpretation and extended care treatment allocation [20]. In our study, we included individuals in the maintenance phase based on participation in at least one prior IVR call, not based on 6-month achievement of behavioral or anthropometric outcomes. This was a practical decision based on the desire to promote overall retention rates. However, further analyses are warranted to account for those individuals who achieved versus did not achieve improvements in the 6-month phase, as these findings could reveal additional insights about the 18-month maintenance of effects. Likewise, within each stratum described above we randomly assigned individuals into the maintenance phase conditions. Regardless of participation level in the IVR component during the initial 6 months, participants were randomized into one of the three conditions (e.g., those with either low or high engagement in the IVR could have been randomized to human-support delivered maintenance calls). While this randomized approach was scientifically justified to test our maintenance hypotheses, other pragmatic options should also be considered in future studies. For example, rather than random assignment, a stepped care model should be considered such that the most effective and least resource intensive maintenance support is provided [69]. Participants in our trial who were adequately engaged with the IVR could have remained in the IVR maintenance condition, whereas those who were less engaged in the IVR and/or failed to achieve outcomes could have been allocated to a human-delivered condition. Evaluating reach, effectiveness and implementation outcomes from a stepped care allocation scheme may also be useful to real-world systems. Finally, from a socio-ecological perspective, additional research is needed to integrate evidence-based individual-level behavioral programs aimed at achieving and maintaining SSB reduction, like SIP*smart*ER, with higher level environmental- and policy-level approaches [12–15]. This multi-level effort is even more critical when extending the focus from SSB behavior change to the long-term improvement of complex chronic health conditions impacted by excessive SSB intake (e.g., obesity, type 2 diabetes, cardiovascular disease, caries and oral health).

## Conclusions

In conclusion, relative to comparison participants, the primary SSB outcome from this RCT was maintained among SIP*smart*ER participants. Brief monthly behavioral based maintenance calls delivered via human support offered no advantage over behavioral based IVR calls, with regard to outcomes, completion or retention rates, or costs. While additional research is certainly warranted, our findings suggest that using IVR to support SSB behaviors offers advantages as an effective, scalable, and affordable maintenance strategy in rural regions.

## Abbreviations

BMI: body mass index
IVR: interactive voice response
SSB: sugar-sweetened beverages
